# High maternal mortality in Jigawa State, Northern Nigeria estimated using the sisterhood method

**DOI:** 10.1186/s12884-017-1341-5

**Published:** 2017-06-02

**Authors:** Vandana Sharma, Willa Brown, Muhammad Abdullahi Kainuwa, Jessica Leight, Martina Bjorkman Nyqvist

**Affiliations:** 1 Abdul Latif Jameel Poverty Action Lab, Massachusetts Institute of Technology, 30 Wadsworth St, Cambridge, MA 02142 USA; 2 Jigawa Ministry of Health, Jigawa, Nigeria; 3 Department of Economics, Williams College, Williamstown, MA USA; 4 Department of Economics, Stockholm School of Economics, Stockholm, Sweden

**Keywords:** Maternal mortality, Sisterhood method, Jigawa, Nigeria, Northern Nigeria, Sub-Saharan Africa

## Abstract

**Background:**

Maternal mortality is extremely high in Nigeria. Accurate estimation of maternal mortality is challenging in low-income settings such as Nigeria where vital registration is incomplete. The objective of this study was to estimate the lifetime risk (LTR) of maternal death and the maternal mortality ratio (MMR) in Jigawa State, Northern Nigeria using the Sisterhood Method.

**Methods:**

Interviews with 7,069 women aged 15–49 in 96 randomly selected clusters of communities in 24 Local Government Areas (LGAs) across Jigawa state were conducted. A retrospective cohort of their sisters of reproductive age was constructed to calculate the lifetime risk of maternal mortality. Using most recent estimates of total fertility for the state, the MMR was estimated.

**Results:**

The 7,069 respondents reported 10,957 sisters who reached reproductive age. Of the 1,026 deaths in these sisters, 300 (29.2%) occurred during pregnancy, childbirth or within 42 days after delivery. This corresponds to a LTR of 6.6% and an estimated MMR for the study areas of 1,012 maternal deaths per 100,000 live births (95% CI: 898–1,126) with a time reference of 2001.

**Conclusions:**

Jigawa State has an extremely high maternal mortality ratio underscoring the urgent need for health systems improvement and interventions to accelerate reductions in MMR.

**Trial registration:**

The trial is registered at clinicaltrials.gov (NCT01487707). Initially registered on December 6, 2011.

## Background

Maternal mortality, defined as the death of a woman during pregnancy or within 42 days after birth, remains a major global health problem [1]. In 2013, there were an estimated 293,000 maternal deaths worldwide, with 99% of these occurring in low income countries [2]. The African continent is disproportionately affected; 17 of the 20 countries with the highest maternal mortality ratios in the world are in Africa [2]. Most maternal deaths are avoidable and the goal of preventing maternal mortality has received increasing attention. Maternal mortality reduction was included as one of the Millennium Development Goals (MDG 5), with the aim of reducing maternal deaths by 75% from 1990 to 2015 [3]. However, progress towards this objective has been slow in many sub-Saharan African countries.

In Nigeria, for example, one of the six countries that together contribute >50% of the total maternal deaths worldwide, reductions in the maternal mortality ratio (MMR; number of maternal deaths per 100,000 live births) have been inconsistent. The most recent national data estimates the MMR for Nigeria to be 576 deaths per 100,000 live births (95% CI: 500–652) [4]. Previous estimates of MMR in Nigeria ranged from 608 deaths per 100,000 live births (95% CI: 372–946) in 2008, to 473 deaths per 100,000 live births (95% CI: 360–608) in 1990 [5].

Maternal mortality is difficult and complex to measure especially in the settings where the highest burden exists. Data on the number of deaths of women of reproductive age, their pregnancy status at the time of death and the medical cause of death is required for accurate measurement. This can be particularly difficult to obtain in low-income settings where vital statistics are often incomplete or do not exist. Estimates are frequently based on hospital data, which often do not reflect the maternal risk within communities [6]. Community based studies using direct estimation to measure maternal mortality face numerous challenges including large sample sizes required to produce reliable results, and the fact that most deaths occur at home and follow up is therefore time consuming and costly.

Indirect methods for measuring maternal mortality have been developed to provide practical, less expensive alternatives for estimating the MMR in settings were data on vital events are not routinely collected or are unreliable. The Sisterhood Method for estimating MMR, which involves collecting data on maternal deaths among sisters of respondents, is an ideal method for such settings because it requires a smaller number of respondents than cohort studies, and data collection is quick and relatively simple [7]. The Sisterhood Method has been validated and applied successfully in a number of countries in Africa and Asia [8–11]. However, this method does not provide a current estimate of MMR for the year the survey is conducted and cannot be used to measure trends in the short term [12]. Longer term trends in MMR, however, have been assessed using the Sisterhood Method [13].

In Nigeria, there is significant variation in the MMR between regions in the country, with the highest ratios in the north [14–19]. The extremely poor health outcomes for mothers in Northern Nigeria are linked to factors such as weak health infrastructure, low literacy and large distances from health facilities [20]. Skilled birth attendance (SBA) is extremely low with only 13% of women delivering with skilled personnel [21]. In addition, vital event registration is virtually non-existent in the north and most maternal deaths occur at home and are unreported. This suggests that maternal mortality data collected at the facility level may not be accurate, and community-based approaches to assess MMR such as the Sisterhood Method may be essential.

Two previous studies estimating MMR in Northern Nigeria using the Sisterhood Method reported an MMR of 1,049 maternal deaths per 100,000 live births (95% CI: 1021–1136) in Zamfara State, and 1,271 maternal deaths per 100,000 live births (95% CI: 1152–1445) across Zamfara, Jigawa, Katsina and Yobe States [18, 19]. The second study was not powered for state-level estimates, and thus, to date, no reliable population-based estimate of MMR for Jigawa State in the northeastern region of the country is available. This lack of reliable state-level data poses a challenge to the planning and implementation of safe motherhood programs in the state. The primary objective of this study, therefore, was to estimate lifetime risk of maternal death and calculate the MMR in Jigawa State, Northern Nigeria using the Sisterhood Method.

## Methods

This study utilized baseline data from an ongoing cluster-randomized controlled trial of community-based interventions to reduce maternal mortality in Jigawa State, Northern Nigeria. The trial is being implemented by the Abdul Latif Jameel Poverty Action Lab (J-PAL) in partnership with the Planned Parenthood Federation of Nigeria (PPFN) to evaluate the impacts of three separate interventions: 1) training local women as Community Resource Persons (CoRPs) who provide education and referrals to pregnant women and their families, 2) the CoRPs program plus distribution of safe birth kits to pregnant women, 3) the CoRPs program plus community dramas to change social norms around maternal health.

### Study area

Jigawa State, located in North-Western Nigeria, had a population of 4.3 million inhabitants during the 2006 census [22]. The state is divided into 27 Local Government Areas (LGAs), with 80% of the population residing in rural areas.

### Study design and sampling method

LGAs were included in the study if they had a Primary Health Center (PHC) that had received the government’s Midwives Service Scheme (MSS) and was thus providing 24-h maternity care at the time of the study start. Of the 27 LGAs in Jigawa State, the following 3 were ineligible for participation in the study: Jahun, Hadejia, and Gumel. Using a list of settlements and their populations in each LGA, 96 clusters of settlements with an average population of approximately 3,000 people per cluster were randomly selected. Each of the 96 population clusters were mapped during a partial census, and 15% of households were randomly selected to participate in the baseline study. Wives of the household head or female household heads in sampled households who were of reproductive age (between 15 and 49 years of age) were eligible to be interviewed. In cases where a household had more than one eligible respondent, one respondent was randomly selected using a randomization table. Households that declined to participate were replaced with back-up households.

### Data collection

The baseline survey was conducted between December 2011 and May 2012. Data were collected from a total of 7,069 women of reproductive age using a structured questionnaire translated to Hausa, the local language, focused on maternal and child health. Female, Hausa speaking interviewers from the study areas collected data using smart phones programed with Open Data Kit electronic data collection software. The questionnaire also included the four standard indirect Sisterhood Method questions in order to estimate MMR in the state. The specific questions included were: 1) How many sisters (born to the same mother) have you ever had who reached the age of 15 (including those who are now dead)?, 2) How many of these sisters are alive now?, 3) How many of these sisters are dead?, 4) How many of the dead sisters died during pregnancy, labor or within 42 days after delivery?. Interviewers checked that the sum of questions two and three was equal to the total in question one.

### Data analysis

Data were analyzed using Stata 13.1 (Stata Corp, College Station, TX). For each 5-year age group, the number of sisters exposed to the risk of maternal death and the duration of their exposure was calculated by multiplying the number of sisters by an age-specific adjustment factor. The lifetime risk of maternal death (LTR) was calculated by dividing the total number of maternal deaths by the estimated total number of sisters exposed. An average estimate of the total fertility rate (TFR) in Jigawa state of 6.7 was obtained from the 2011 Multiple Indicator Cluster Survey [23]. The formula used to calculate MMR from the LTR was: MMR = 100,000 × (1- [1-LTR]^(1/TFR)^) [6]. Ninety-five percent confidence intervals for MMR were calculated using the method of Hanley et al. [24]. The time period to which the estimate refers was calculated through the following equation: T = Σ (T(i)*B(i))/ΣB(i), where T = the point time location of the global estimate, T(i) = the time location of the estimate for each age group and B(i) = the exposing units of each age group [6].

### Ethics

Verbal informed consent was obtained from all respondents. Ethical approval was obtained from the Massachusetts Institute of Technology (MIT) and the Jigawa State Operations Research Advisory Committee (ORAC). The trial is registered at clinicaltrials.gov (NCT01487707).

## Results

A total of 7,069 respondents completed the survey. The average age of respondents was 27.9 years (SD = 8.5) (Table 1). The majority of respondents had no formal schooling (64.8%), and only 9.6% were literate. Almost all respondents were Muslim (99.9%), and 30.5% reported they were in polygamous households. Respondents reported on average 4.0 births (SD = 3.1).

**Table 1:** Characteristics of respondents in study sample

Variable	*N* (%)
Age (years)	
15–19	1397 (19.8)
20–24	1476 (20.9)
25–29	1328 (18.8)
30–34	1039 (14.7)
35–39	915 (12.9)
40–44	646 (9.1)
45–49	268 (3.8)
Attended School	
Yes	2488 (35.2)
No	4581 (64.8)
Literate	
Yes	675 (9.6)
No	6394 (90.5)
Religion	
Muslim	7060 (99.9)
Catholic	5 (0.07)
Other Christian	4 (0.06)
Polygamous Household	
Yes	2159 (30.5)
No	4910 (69.5)
Latrine Ownership	
Yes	5803 (82.1)
No	1266 (17.9)
Facility based delivery (for deliveries in last 24 months)	
Yes	319 (8.5)
No	3423 (91.5)

The 7,069 respondents reported 10,957 maternal sisters who survived past the age of 15 years of which 1,026 (9.4%) were reported dead. Of the 1,026 deaths, 300 (29.2%) occurred during pregnancy, childbirth or within 42 days after delivery. Table 2 shows the sisters’ vital status by 5-year age groups and the LTR of maternal death for the entire cohort. The total lifetime risk of maternal death was 6.6% or 1 in 15. Using a TFR of 6.7 for Jigawa State, the estimated MMR for the study areas was 1,012 maternal deaths per 100,000 live births (95% CI: 898 – 1,126). The approximate time reference for the MMR estimate was the year 2001 corresponding to 11.0 years before the interviews.

**Table 2:** Maternal mortality estimate using the sisterhood method for the reference period 2001 using in Jigawa State, Nigeria

Age group of respondent (yrs)	Number of respondents		Number of sisters survived age > 15 yrs		Number of sisters who died		Number of maternal deaths		Age-specific adjustment	Sisters exposed (B)	Lifetime total risk
	k		e		C		r		f	β = ef	Q = r/β
	n	%	n	%	n	%	n	%		n	
15–19	1397	19.76	1786	16.30	143	13.94	30	10.00	0.107	191	0.157
20–24	1476	20.88	2234	20.39	200	19.49	64	21.33	0.206	460	0.139
25–29	1328	18.79	2092	19.09	157	15.30	45	15.00	0.343	718	0.063
30–34	1039	14.70	1718	15.68	158	15.40	43	14.33	0.503	864	0.050
35–39	915	12.94	1632	14.89	177	17.25	61	20.33	0.664	1084	0.056
40–44	646	9.14	1093	9.98	146	14.23	44	14.67	0.802	877	0.050
45–49	268	3.79	402	3.67	45	4.39	13	4.33	0.900	362	0.036
Total	7069	100.0	10957	100.0	1026	100.0	300	100.0		4555	0.066

LGA level analysis in Table 3 showed variability in the proportion of mortality among sisters attributed to maternal causes. While on average 29.2% of deaths to sisters were related to pregnancy and childbirth, in some LGAs such as Sule Tankarkar, Gwiwa, and Kiyawa this figure was 50% or higher.

**Table 3:** Reported vital status of sisters by Local Government Area (LGA) in Jigawa state, Nigeria

LGA	Number of respondents	Number of sisters survived >15 yrs	Number of sisters died		Number of maternal deaths	
			n	%	n	%
Dutse	307	387	39	10.1	7	17.9
Gwaram	294	539	43	8.0	15	34.9
Miga	355	457	33	7.2	10	30.3
Birniwa	261	293	29	9.9	11	37.9
Kaugama	183	312	29	9.3	14	48.3
Mallam Madori	257	244	33	13.5	16	48.5
Babura	288	569	88	15.5	14	15.9
Gagarawa	278	567	80	14.1	15	18.8
Garki	259	482	65	13.5	18	27.7
Maigatari	223	396	48	12.1	11	22.9
Ringim	351	508	53	10.4	16	30.2
Roni	323	673	57	8.5	9	15.8
Birnin Kudu	355	710	56	7.9	19	33.9
Buji	330	613	51	8.3	19	37.3
Kiyawa	347	402	37	9.2	20	54.1
Auyo	289	494	32	6.5	11	34.4
Guri	315	607	39	6.4	19	48.7
Kafin Hausa	284	385	53	13.8	11	20.8
Kirikasamma	313	377	38	10.1	7	18.4
Gwiwa	271	389	18	4.6	10	55.6
Kazaure	317	534	32	6.0	10	31.3
Sule Tankarkar	257	224	8	3.6	4	50.0
Taura	337	526	56	10.6	12	21.4
Yankwashi	275	269	9	3.3	2	22.2
Total	7069	10957	1026	9.4	300	29.2

In order to obtain an estimate of MMR for the most recent period, the analysis in Table 2 was replicated for women below 30 years of age. A total of 4,201 respondents reported 6,112 sisters of which 500 (8.2%) were reported dead. Of those 500 dead sisters, 139 (27.8%) were due to maternal causes. The total lifetime risk of maternal death for women under 30 years was 10.2% or 1 in 10. The MMR estimate for this age group was 1,586 maternal deaths per 100,000 live births (95% CI: 1,326 -1,849), corresponding to a period of 7.3 years before the survey.

## Discussion

This study reports a maternal mortality ratio of 1,012 maternal deaths per 100,000 live births in Jigawa State, Northern Nigeria. This is significantly higher than the most recent national estimates, also conducted using the Sisterhood Method, suggesting a MMR of 576 maternal deaths per 100,000 live births (time reference of 2006) and highlights the extremely high burden of maternal mortality in the northern part of the country [4]. The results are consistent with two other studies of MMR estimated by the Sisterhood Method in Northern Nigeria. The first study collected data in 2009 and reported an MMR of 1,049 maternal deaths per 100,000 live births (95% CI: 1021–1136) in Zamfara State [18]. The second study, conducted in 2011, estimated a MMR of 1,271 maternal deaths per 100,000 live births (95% CI: 1152–1445) in four states in Northern Nigeria (Zamfara, Jigawa, Katsina, Yobe) [19]. While that study included some data from Jigawa state, it was not adequately powered for state level estimates of MMR. Furthermore, data in that study was collected in specific project sites and may not have been representative of other areas within the states. Our results therefore provide the first evidence-based estimation of MMR in Jigawa State. Our sampling procedures involved randomly selecting households within randomly sampled communities spanning 24 of 27 LGAs within Jigawa, suggesting the results are representative at the state level.

Our study also found variation in maternal mortality by LGA. Mallam Madori, Kiyawa, Gwiwa, Sule Tankarkar, Kaugama and Guri in particular had extremely high proportions of deaths among sisters that were attributed to maternal causes. Many of these areas are characterized as being remote with larger distances to health facilities. Similar to other reports, the highest burden of maternal mortality in our study is in the youngest group of respondents. Others have postulated that this phenomenon is linked to low age at marriage and early childbearing [18, 25]. However, in our data, age at death amongst respondents’ sisters was not available and this explanation could not be explored. However, since these deaths would have occurred more recently, information provided by these younger respondents is likely to be more reliable than reports from the older cohorts. Thus, an alternative explanation for the higher maternal mortality in the younger group could be reduced misreporting and recall bias.

This study has several limitations. First, the reported MMR refers to a period of approximately 11 years before data collection, or the year 2001. It is not possible to estimate MMR at the time of data collection within the study communities using this methodology. Second, data on the location of the sisters was not available, and we used the village of the respondent as a proxy for the village of the sisters. This approach is consistent with other research using the Sisterhood Method [4, 18, 19, 25]. Third, LGAs included in this study all had at least one PHC that was receiving the government’s MSS at the start of the study. It is possible that the MMR is higher in those LGAs that were excluded from the study, however other data collected (not shown) demonstrate that substantial health systems challenges persist within the MSS facilities contributing to high mortality. Fourth, while the sample was representative at the state level, the results may be less generalizable to other areas in the region due to differences in health services delivery and access and other differences. Finally, misreporting of number of sisters and number of deaths in sisters who reached reproductive age could have led to under or overestimates of MMR. For example early pregnancy and abortion related deaths could have been misclassified as non-maternal deaths leading to underestimations or respondents could have forgotten the timing of deaths.

Our data show a large number of respondents who reported having no sisters that reached reproductive age. These cases seemed to be clustered in specific LGAs or linked to specific enumerators and were potentially caused by misunderstanding of the question during interviews. However, sensitivity analysis showed virtually no change in the estimated MMR if data from these particular LGAs or enumerators were excluded from the analysis. In addition, for those respondents who reported having sisters that reached reproductive age, the average number of sisters per respondent was 2.6, which is consistent with a TFR of 6.7 and with other studies. This suggests that these respondents’ data were reliable.

## Conclusions

Our findings show an extremely high maternal mortality ratio of 1,012 maternal deaths per 100,000 live births (95% CI: 898–1,126) in Jigawa State, Northern Nigeria. This underscores the need to strengthen obstetric care in the area and for expansion of safe motherhood interventions to accelerate reductions in maternal deaths.

## Abbreviations

CoRPs: Community resource persons
J-PAL: Abdul Latif Jameel Poverty Action Lab
LGA: Local government area
LTR: Lifetime total risk of maternal mortality
MDG: Millennium Development Goal
MIT: Massachusetts Institute of Technology
MMR: Maternal mortality ratio
MSS: Midwives Service Scheme
ORAC: Jigawa State Operations Research Advisory Committee
PHC: Primary health center
PPFN: Planned Parenthood Federation of Nigeria
SD: Standard deviation
TFR: Total fertility rate
